# Melan-A-Positive Granular Cell Tumor With Extensive Intraneural and Perineural Spread: A Rare Case Report

**DOI:** 10.7759/cureus.59903

**Published:** 2024-05-08

**Authors:** Svetlana Bobkova, Eli Oldham, Igor Shendrik

**Affiliations:** 1 School of Biomedical Science, Oklahoma State University Center for Health Sciences, Tulsa, USA; 2 Office of Medical Student Research, Oklahoma State University Center for Health Sciences, Tulsa, USA; 3 Dermatopathology Section, Regional Medical Laboratory, Tulsa, USA; 4 Department of Dermatology, University of Oklahoma College of Medicine, Tulsa, USA

**Keywords:** granular cell tumor, dermatopathology, hmb-45, melan-a, inhibin-α, s-100, immunohistochemistry, tumor

## Abstract

Cutaneous granular cell tumors (GCTs) are rare tumors that typically exhibit benign clinical behavior and are likely of Schwann cell origin. Some histologic and immunohistochemical variants of GCTs may present challenges due to infiltrative growth patterns, perineural invasion, and expression of Melan-A. In this case report, we present a 27-year-old male who had previously been diagnosed with a typical GCT on the back a few years ago. The current biopsy from the proximal palm demonstrated a cytologically similar tumor with extensive perineural spread and notable positivity for Melan-A. Although uncommon, these features are consistent with the histological appearances of GCTs. The current views on the histogenesis of GCTs, clinical associations, differential diagnosis with melanoma, and histological criteria for malignant GCTs are discussed. A panel of immunohistochemical stains, including Inhibin-α and preferentially expressed antigen in melanoma (PRAME), is proposed for use in rare instances of Melan-A-positive GCTs.

## Introduction

Granular cell tumors (GCTs) are a rare neoplasm generally believed to be of Schwann cell origin [1]. These tumors typically have a benign clinical course but may occasionally demonstrate malignancy [1]. Some variants of GCTs may pose a diagnostic challenge due to infiltrative growth patterns, perineural invasion, and Melan-A expression [2]. In this case report, we describe a 27-year-old male previously diagnosed with a GCT on the back, who presented with a similar tumor on the proximal palm, exhibiting extensive perineural spread and marked positivity for Melan-A. The usefulness of an immunohistochemical panel consisting of Inhibin-α, preferentially expressed antigen in melanoma (PRAME), and HMB-45 stains for diagnosing GCTs is discussed.

## Case presentation

**Figure 1:**
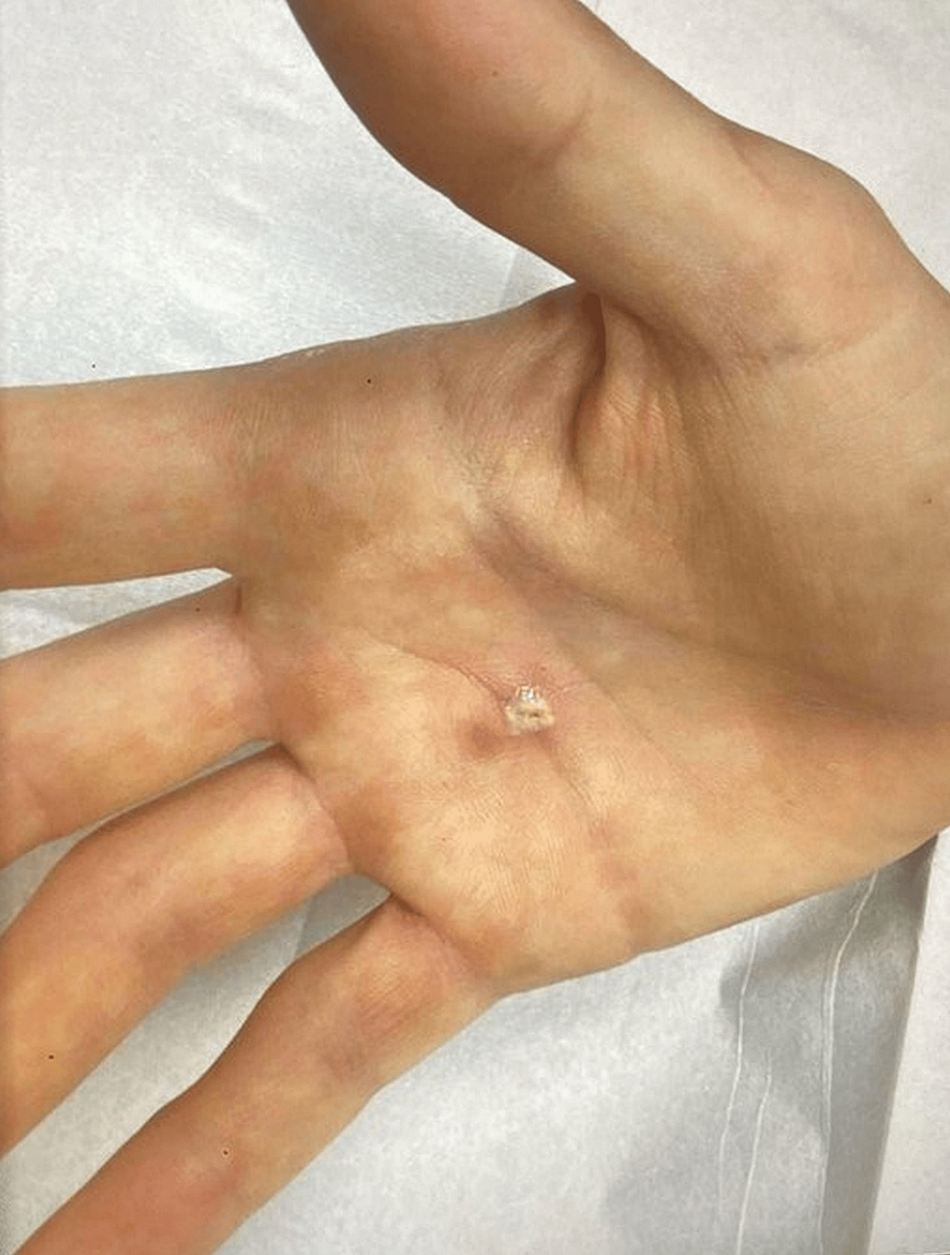
Nodule on the patient's right proximal palm prior to excision.

A 27-year-old male sought medical attention due to concern about two tender subcutaneous nodules that had been present for several months. One nodule was located on the dorsal aspect of the left second digit, while the second nodule was on the proximal palm of the right hand (Figure 1). The nodules were both approximately 0.5 cm in diameter, firm, and non-fluctuant. The clinical differential diagnosis included cyst, neuroma, and fibromatosis.

The patient's personal history was notable for a histologically confirmed benign GCT on the back that had been surgically excised a few years prior and showed no recurrence. The previous GCT was immunohistochemically tested, demonstrating a classic profile with positivity for S-100 and negative staining for Melan-A. The patient had no other significant past medical history or family history of similar lesions, and he demonstrated no diagnostic features of genetic syndromes.

A biopsy of the left dorsal second digit revealed a primary GCT with classic histological and immunohistochemical appearance, similar to the histologic appearance of the patient’s previous back nodule.

**Figure 2:**
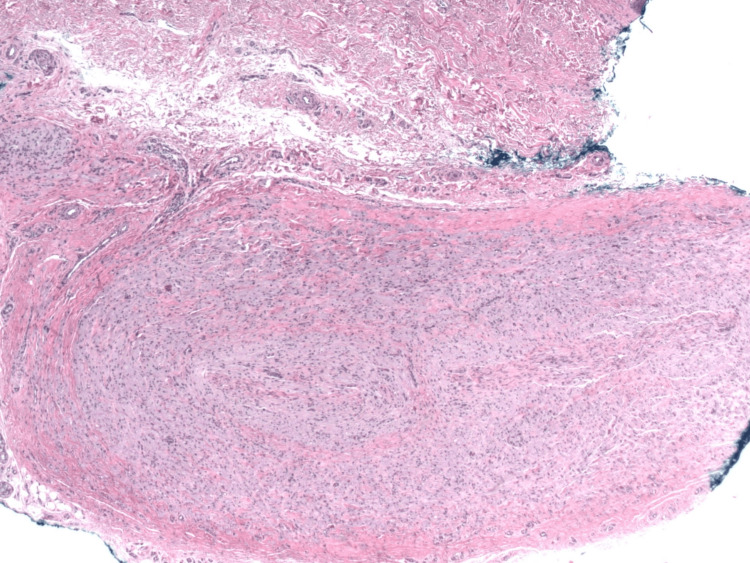
Biopsy of the right palm showing a deeply seated nodule horizontally aligned with neural twigs (H&E, original magnification ×2).

**Figure 3:**
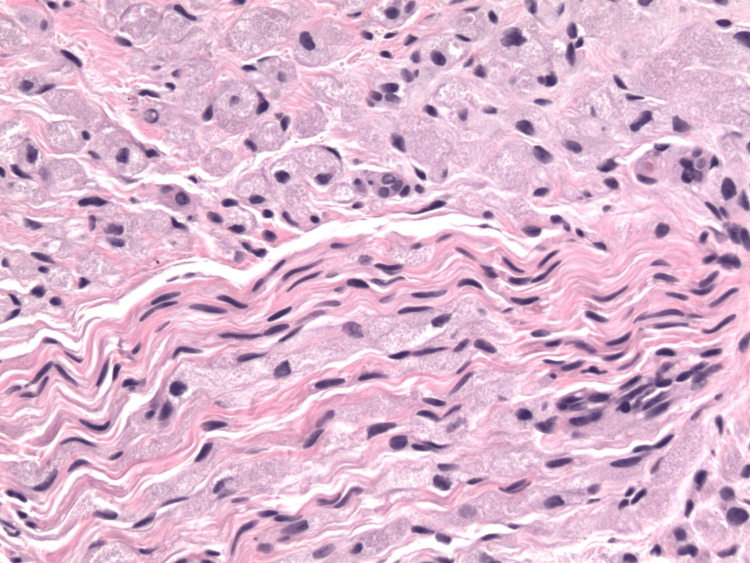
Biopsy of the right palm showing a granular tumor with extensive perineural and intraneural infiltration (H&E, original magnification ×20).

**Figure 4:**
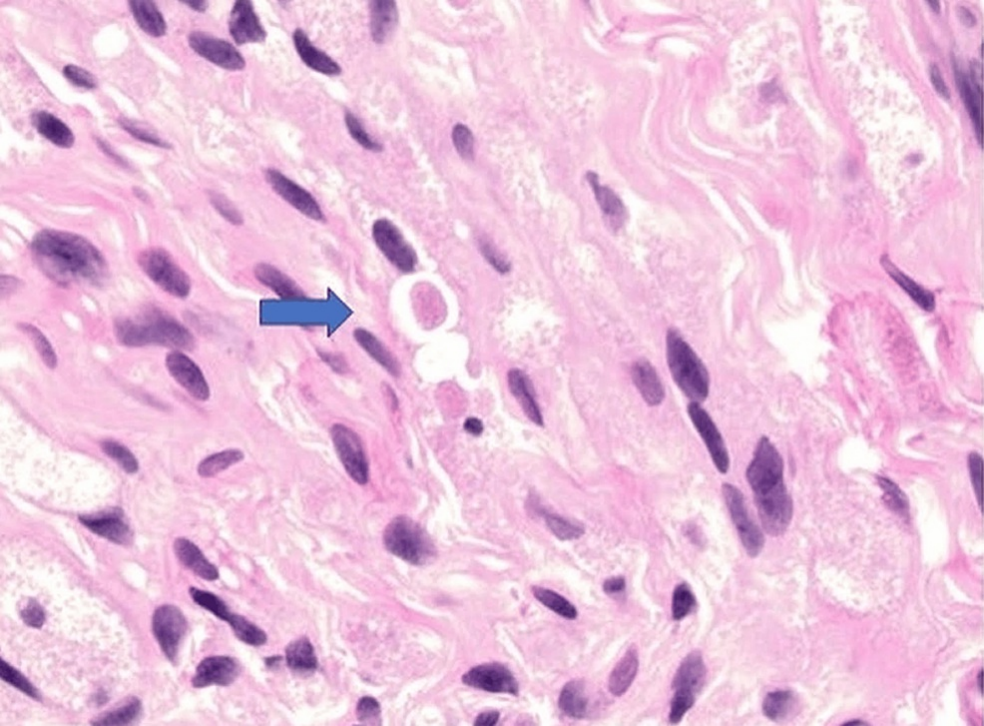
A, GCT with Pustulo-ovoid bodies of Milian (arrow) (H&E, original magnification ×40). GCT: Granular cell tumor.

**Figure 5:**
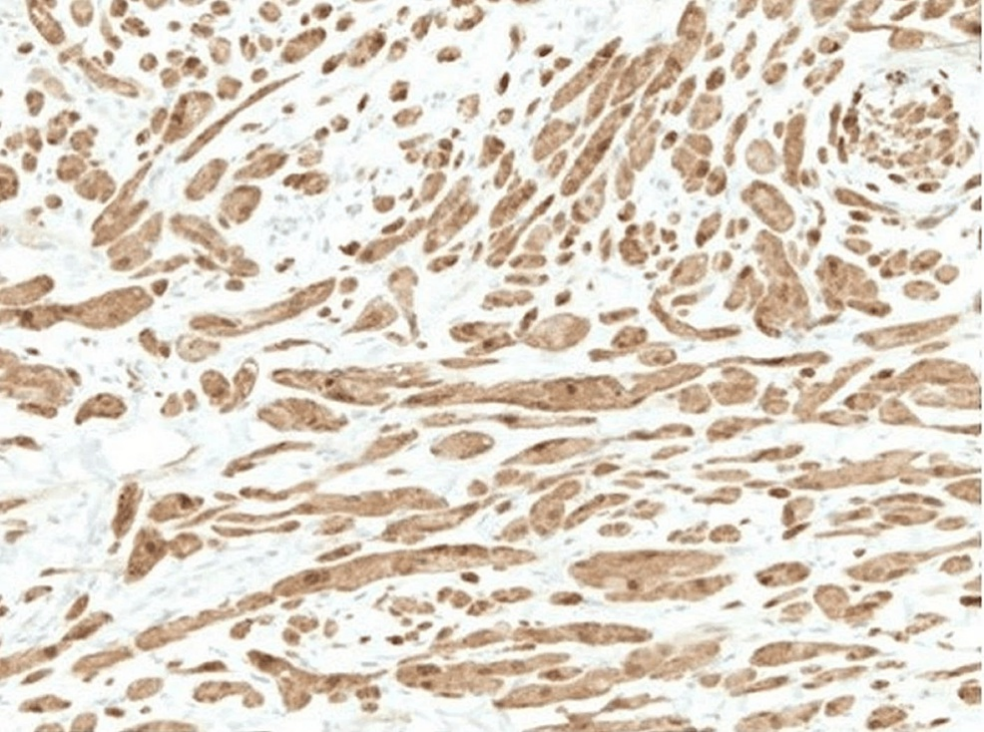
Immunohistochemical stain with S-100 (original magnification ×10).

**Figure 6:**
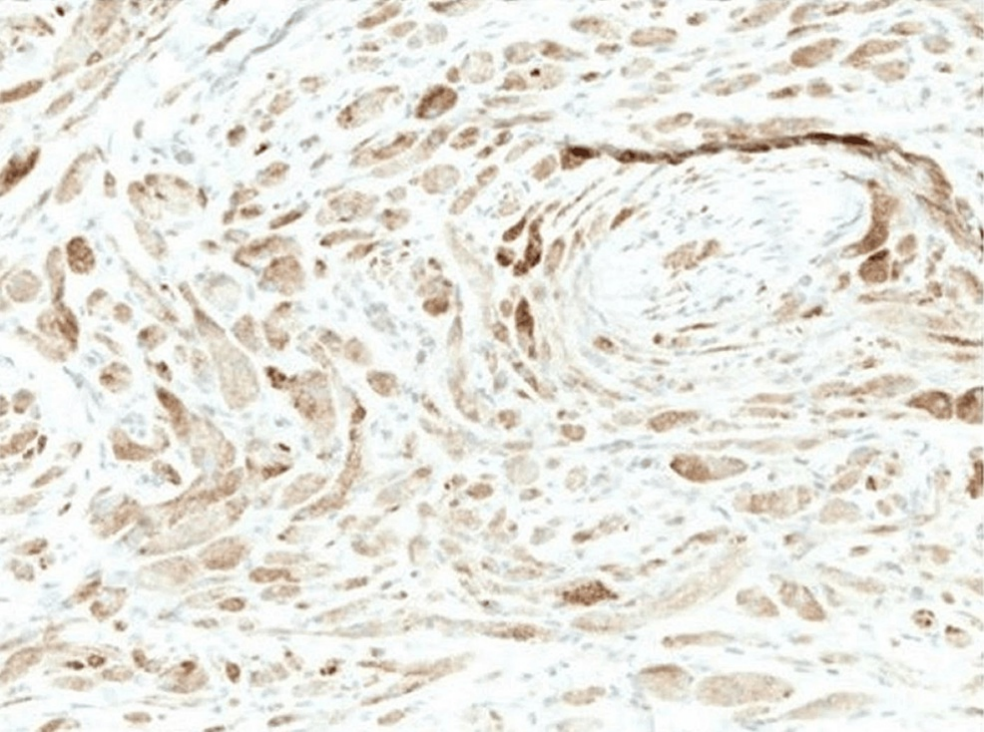
Immunohistochemical stain with Inhibin-⍺ (original magnification ×10).

**Figure 7:**
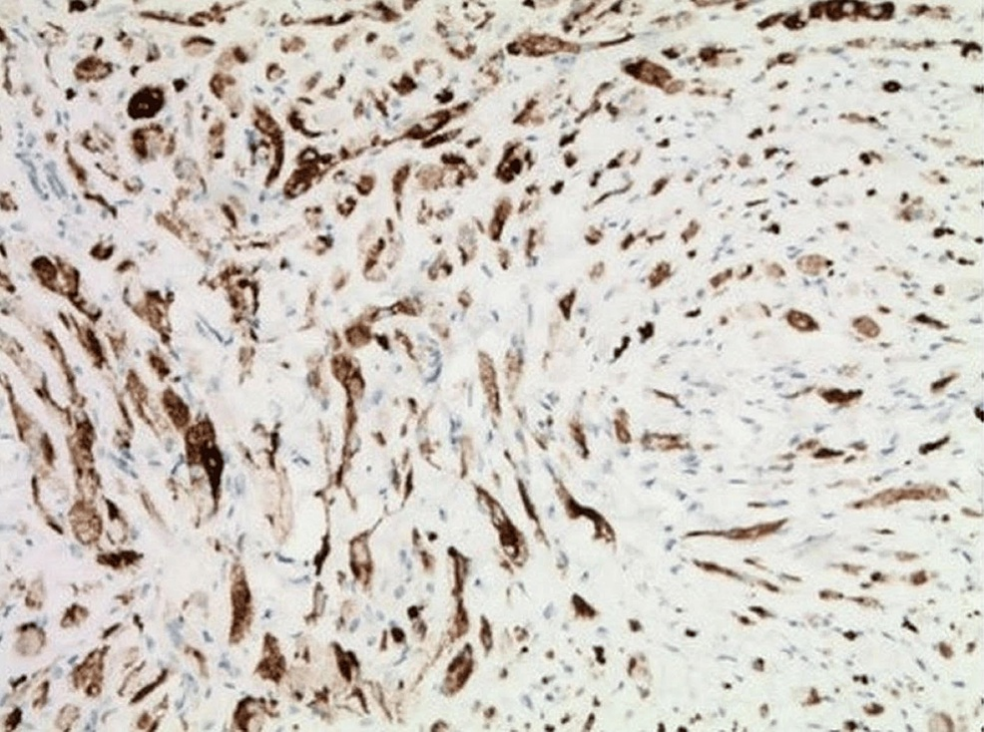
Immunohistochemical stain with Melan-A (original magnification ×10).

The biopsy of the palm nodule revealed a primary GCT with uncommon histological and immunohistochemical features. This palm GCT demonstrated a horizontally oriented, circumscribed, elongated lesion (Figure 2) composed of spindle and polygonal cells with abundant eosinophilic granular cytoplasm and round nuclei. The tumor showed prominent intraneural and perineural spread, with splitting of neural bundles (Figure 3). The so-called Pustulo-ovoid bodies of Milian (Figure 4), defined as an eosinophilic globule surrounded by a clear halo, were present. The lesion was not mitotically active. Immunohistochemical evaluation revealed the tumor to be positive for S-100 (Figure 5), Inhibin-α (Figure 6), and Melan-A (Figure 7), while negative for HMB-45 and preferentially expressed antigen in melanoma (PRAME).

The palm nodule was diagnosed as a benign cutaneous GCT, and the original excision of the lesion was determined to be an adequate treatment for the patient. The excised GCT exhibited focal involvement at the inked surgical margins, but the benign nature of the tumor warranted no further interventions. Further, no tumor recurrence was seen after 12 months.

## Discussion

GCTs are an uncommon lesion that can occur in any anatomical location, but they most commonly affect extraneural sites, notably the skin or subcutaneous tissue [3]. While it is widely accepted that GCTs are of Schwann cell origin [3], some researchers have postulated the possibility that GCTs originate from undifferentiated mesenchymal cells that acquire partial Schwannian differentiation [4]. Some benign examples may cause concern for malignancy due to focal nuclear pleomorphism, prominent nucleoli, perineural invasion, and scattered mitoses. Conversely, many benign and malignant neoplasms, including dermatofibroma, dermatofibrosarcoma protuberans, cellular neurothekeoma, leiomyoma, leiomyosarcoma, basal cell carcinoma, angiosarcoma, and melanoma, may show granular cell change and thus may be considered in the morphologic differential diagnosis of GCT [1].

GCTs can be either solitary or multiple. Some, but not all, cases of multiple GCTs may be associated with an underlying genetic syndrome such as PTEN hamartoma tumor syndrome, neurofibromatosis type 1, Noonan syndrome, and LEOPARD syndrome [2]. GCTs frequently harbor loss-of-function mutations in the vacuolar H+-ATPase (V-ATPase) components, most commonly ATP6AP1 and ATP6AP2 [5]. Multifocal GCTs within a given patient are molecularly distinct, while paired primary and metastatic histologically malignant GCTs show identical mutations, allowing confirmation of the primary-metastatic relationship [5].

While genetic studies of GCTs are a rapidly developing field, the number of studies is limited, leaving immunohistochemistry as a primary method of diagnosis, with S-100 being a defining marker of this tumor [6]. Although strong expression of S-100 protein allows the exclusion of most cytologically similar tumors, the differential diagnosis with malignant melanoma can be challenging due to considerable immunophenotypic overlap between GCT and melanoma, with both tumors expressing S-100 protein and NKIC3, and more than half of melanomas expressing CD68 and NSE [1]. Moreover, the rare expression of Melan-A by GCT, which has been demonstrated in approximately 5% of cases, can further complicate the differentiation between GCT and melanoma [1]. Recent studies have proposed Inhibin-α subunit and calretinin as sensitive markers for GCT, which are rarely expressed in melanoma [1].

Although the most definitive criterion for distinguishing benign GCT from malignant GCT is the presence of metastasis, in 1998, Fanburg-Smith JC et al. subdivided GCT into 'benign,' 'atypical,' and 'malignant' based on six histologic criteria: nuclear pleomorphism, tumor cell spindling, vesicular nuclei with large nucleoli, increased nuclear-to-cytoplasmic ratio, necrosis, and increased mitotic rate (> 2 mitoses/10 HPF) [7]. Tumors were considered 'benign' if no or only focal nuclear pleomorphism was seen, 'atypical' when one or two features were present, and 'malignant' if three or more features were observed [7].

More recently, Nasser et al. proposed a diagnostic approach to define GCT of uncertain malignant potential, which depends only on necrosis and/or mitoses as the most reproducible criteria [8]. While large series with long-term follow-up are not available, local recurrence of incompletely excised tumors is approximately 20%, while metastatic disease is seen in approximately 25% of histologically malignant tumors by Fanburg-Smith JC et al. criteria [6].

The current case presented a diagnostic challenge due to extensive perineural and intraneural tumor infiltration, as well as positivity for Melan-A and focal spindle morphology. However, the overall histological appearance of the lesion was consistent with benign GCT, and additional staining for other markers commonly used in the diagnosis of melanoma, such as HMB-45 and PRAME, was negative. This combination of histologic and immunohistochemical features is rare but allowed for a diagnosis of benign GCT in this case. The current case was also immunohistochemically different from the previously diagnosed GCT of this patient, further supporting a diagnosis of multifocal primary GCT as opposed to a metastatic process, which would be expected to show identical morphologic and immunohistochemical properties with the previous tumor [5].

Although the use of the novel immunohistochemical marker, PRAME, has not been previously addressed, recent publications have indicated that PRAME is not expressed in GCTs [9]. Therefore, PRAME could be a valuable addition to an immunohistochemical panel used to differentiate Melan-A-positive GCT from melanoma.

Due to recent advances in our understanding of GCT genetics, targeted next-generation sequencing (NGS) and/or comparative Sanger sequencing may be applied to exclude a metastatic process in some cases, which show atypical or malignant morphology or unusual clinical presentations. However, most cases may be resolved on the basis of histologic and immunohistochemical evaluation.

## Conclusions

We report an intriguing case of multiple GCTs, including one tumor with extensive intraneural and perineural involvement and Melan-A positivity in a patient with a prior history of GCT. The presence of Melan-A in a GCT is a rare finding that can create a diagnostic challenge and mimic melanoma. Diagnostic criteria for atypical and malignant GCTs are reviewed. Notably, in our case, the inclusion of PRAME as part of a comprehensive panel of immunohistochemical markers, including S-100, Inhibin-α, Melan-A, and HMB-45, proved beneficial. Our case suggests that PRAME can assist in the differential diagnosis and may be utilized to resolve similar diagnostic dilemmas. We suggest further studies to evaluate PRAME staining patterns in GCT to substantiate the current body of literature.
